# Synthesis of a Cholesteryl-HEG Phosphoramidite Derivative and Its Application to Lipid-conjugates of the Anti-HIV ^5'^TGGGAG^3'^ Hotoda’s Sequence

**DOI:** 10.3390/molecules171012378

**Published:** 2012-10-22

**Authors:** Domenica Musumeci, Daniela Montesarchio

**Affiliations:** Dipartimento di Scienze Chimiche, Università di Napoli “Federico II”, Complesso Universitario di Monte Sant’Angelo, via Cintia 21, I-80126, Napoli, Italy

**Keywords:** phosphoramidite, cholesterol, solid phase synthesis, anti-HIV G-rich oligonucleotides

## Abstract

A novel phosphoramidite derivative of cholesterol, with an ether-linked hexaethylene glycol (HEG) spacer arm, has been obtained through simple and reproducible solid phase modified oligonucleotide synthesis manipulations. This building block and the known phosphoramidite derivative of 3β-(2-hydroxyethoxy)cholesterol have been exploited in standard oligonucleotide synthesis protocols for the preparation of 5'- conjugates of the G-quadruplex-forming ^5^^'^TGGGAG^3^^'^ oligomer, known as the Hotoda’s sequence, to produce new potential anti-HIV agents.

## 1. Introduction

Cholesterol is one of the most common naturally occurring lipids, being an essential component of higher eukaryotic membranes and playing crucial roles in membrane organization, dynamics and function [1,2]. In addition, its properties have been widely studied in a variety of synthetic organic chemistry applications. Investigated for decades, currently conjugation with steroids is one of the major strategies employed to improve the cellular uptake, enzymatic resistance and biodistribution properties of highly polar bioactive species, thus allowing their *in vivo* applications [3,4,5].

**Figure 1:**
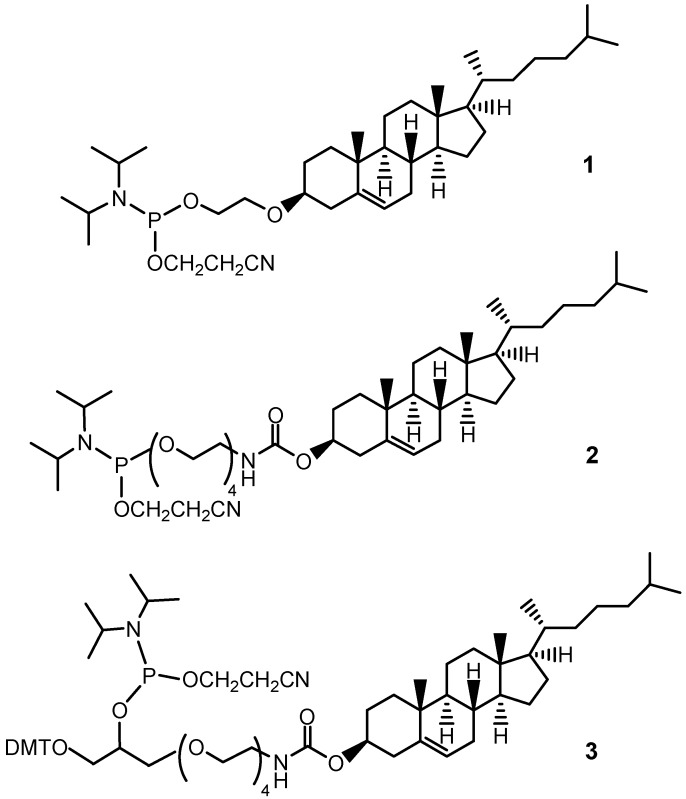
Chemical structures of known phosphoramidite derivatives of cholesterol **1**–**3** [16,18,19].

Since the pioneering works of Letsinger [6,7] and Stein [8], a large number of oligonucleotides have been modified with cholesterol to enhance their biological activity [9]. For such conjugation, disulfide linkages have been efficiently exploited as reversible covalent bonds [10]. For multiple conjugations on the same oligonucleotide sequence, cholesterol has been attached at the 5-position of a suitably protected thymidine monomer [11]. In a more general approach, a specific reporter group can be attached at the 5'- and/or 3'-end of the oligonucleotide sequence through phosphodiester bonds. This strategy, based on the conversion of the conjugating agent into a stable phosphoramidite or H-phosphonate building block, offers a number of advantages, among which the possibility to insert it by an automated solid phase synthetic protocol, exploiting the same procedures used for the oligomer chain assembly on an automated DNA synthesizer, stands out [12,13,14]. In a study by Caruthers *et al.*, cholesterol has been efficiently derivatized as a H-phosphonothioate by simply phosphitylating the 3-OH group, since this steroid, lacking any other nucleophilic moieties, does not require a protection strategy [15]. However, due to the rigidity and high steric hindrance of cholesterol, the insertion of a flexible linker is generally desired for most bioconjugations. One notable phosphoramidite derivative of cholesterol, based on 3β-(2-hydroxyethoxy)cholesterol, has been described by Engels *et al.* (compound **1**, Figure 1), who demonstrated that antisense oligonucleotides conjugated with bile acids or cholesterol showed an enhanced lipophilicity and no significant loss of duplex stability [16]. Though highly appealing for the extreme simplicity of its synthesis, phosphoramidite derivative **1** shows some disadvantages, mainly due to the poor solubility in polar solvents, such as acetonitrile, thus requiring more apolar solvents and therefore *ad hoc* handling solid phase protocols.

In other known cholesteryl phosphoramidite derivatives, spacers longer than the 2-hydroxyethyl group have been introduced on the steroid scaffold, connected through carbamate linkages. Useful cholesteryl phosphoramidite building blocks are commercially available; one compound carries a lipophilic C_6_ linker [17], while two derivatives (compounds **2** [18] and **3** [19], Figure 1) bear a tetraethylene glycol (TEG) linker. These compounds have been exploited in a number of research applications, including biophysical studies on lipid-conjugated oligonucleotides [20,21,22,23]. Interestingly, it has been demonstrated that when the cholesteryl-TEG moiety was covalently bound to an oligonucleotide, the resulting lipophilic DNA molecules inserted spontaneously into lipid membranes without altering their structure, thus significantly differing from the behaviour of pure cholesterol [23].

If for antisense and antigene oligonucleotides the positive contribution of cholesterol as a conjugating agent has been well established [8,10,12,13,15,16], its efficacy in improving the pharmacokinetic profile of G-quadruplex-based aptamers has been only scarcely investigated. A relevant aspect of cholesterol is its steric hindrance, which may be detrimental to G-quadruplex formation, particularly in the case of tetramolecular parallel complexes. In this respect Wolfe and Goodchild demonstrated that the anti-HIV activity of G-rich oligonucleotides can be dramatically influenced, either positively or negatively, by the presence of cholesteryl groups, with enhanced bioactivity only if the hydrophobic groups are covalently attached at positions distant from the G-quartets [24].

In selecting a cholesteryl derivative with optimal features for terminal modifications of G-quadruplex-forming oligonucleotides, two main issues have thus to be considered: the chemical stability of the linker, and its flexibility and length. Indeed, the presence of a cholesteryl residue at one oligonucleotide extremity generates a very hydrophobic micro-environment in the proximity of the sequence to which it is linked. In many cases, especially for short, G-rich oligomers with high propensity to self-aggregate, the hydrophilic/lipophilic domains balance may be unfavourable in terms of water affinity. Thus, long reaction times are necessary for the final aq. ammonia deprotection and water solubility problems for the detached oligonucleotides may arise. In principle, upon prolonged basic treatments at high temperatures, carbamate linkages can be hydrolyzed to a not completely negligible percentage. For these conjugations, therefore, stable and long, hydrophilic linkers are desirable, so that water solubility problems are minimized and hydrophobicity-driven cholesterol assemblies do not hamper, for steric reasons, the correct G-tetrad formation.

**Figure 2:**
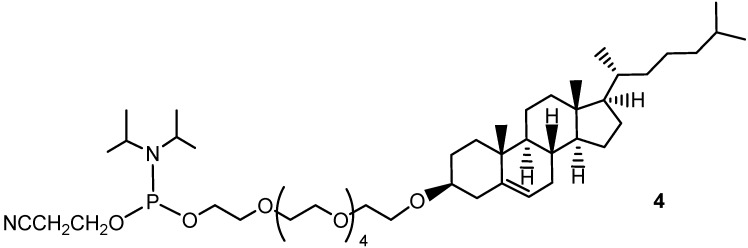
Chemical structure of the herein synthesized phosphoramidite derivative **4**.

Aiming at achieving a pretty stable chemical connection between cholesterol and the terminal oligonucleotide OH groups, with a longer and more polar spacer than TEG, we have therefore designed novel phosphoramidite derivative **4** (Figure 2), in which a hexaethylene glycol (HEG) chain is attached to the 3-OH group of cholesterol through an ether linkage.

To demonstrate its full applicability, we have exploited this building block to functionalize the d(^5^^'^TGGGAG^3^^'^) sequence in parallel with cholesteryl derivative **1**. In the late 90s, Hotoda and coworkers found that short G-rich oligonucleotides, and particularly the sequence d(^5^^'^TGGGAG^3^^'^), if carrying bulky aromatic groups at the 5' end, exhibited potent anti-HIV activity [25,26,27]. Structure-activity relationship investigations on these compounds indicated that G-quadruplex formation was essential for the antiviral activity [25], and large aromatic substituents at the 5'-end favored the G-quadruplex assembly process, from both a thermodynamic and kinetic point of view [28]. Improved anti-HIV potency has been discovered in novel hybrid oligonucleotides carrying the G-quadruplex forming d(^5'^TGGGAG^3'^) sequence, conjugated with different groups at the 3' or 5'-end through phosphodiester bonds, synthesized *via* a fully automated, on-line phosphoramidite-based solid phase strategy [29,30].

**Figure 3:**
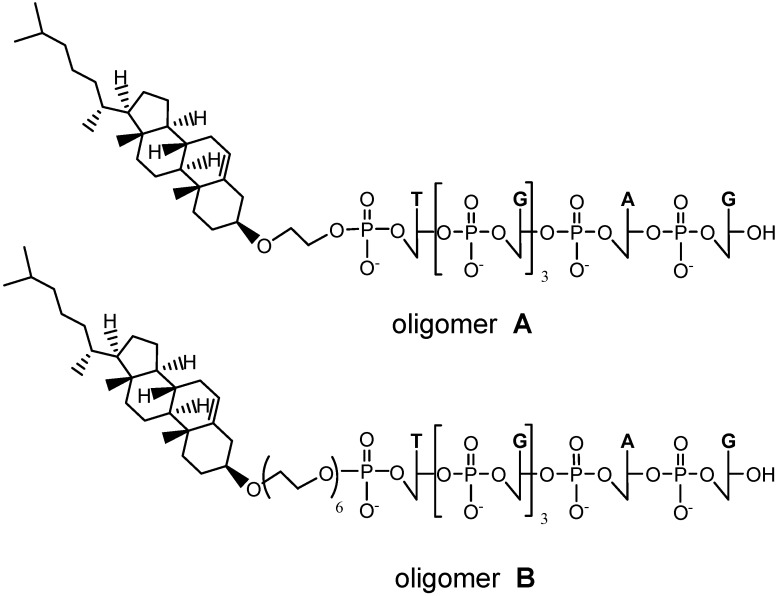
Chemical structure of the 5'-cholesteryl conjugates of Hotoda’s sequence **A** and **B**.

In the framework of a wide research program for the search of antiviral G-quadruplex-based aptamers with optimal features for *in vivo* studies, two novel cholesteryl-conjugated derivatives of the Hotoda’s sequence (**A** and **B**, Figure 3) are here described.

## 2. Results and Discussion

**Scheme 1:**
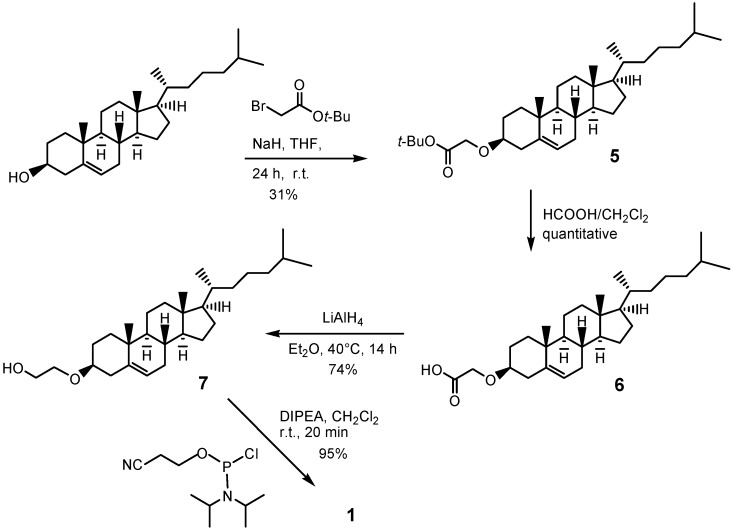
Synthesis of phosphoramidite derivative **1**.

Cholesteryl phosphoramidite **1**, used for the synthesis of 5'-conjugated oligonucleotide **A**, was prepared by exploiting the synthetic route depicted in Scheme 1. In this procedure, cholesterol was first converted into acetic acid derivative **6** through a simple scheme, already used by some of us for HEG and TEG modifications [31,32]. To this end, the starting alcohol was condensed with *tert*-butyl bromoacetate in the presence of NaH giving ester **5**; then the *tert*-butyl group was removed by treatment with formic acid in CH_2_Cl_2_, furnishing the desired carboxylic acid **6**. This was then reduced, by treatment with LiAlH_4_ in diethyl ether at reflux, to alcohol **7**, which was successively reacted with chloro-(2-cyanoethoxy)(*N*,*N*-diisopropylamino)phosphine in the presence of DIPEA, smoothly giving target compound **1**, obtained in 22% overall yield for the four reaction steps.

**Scheme 2:**
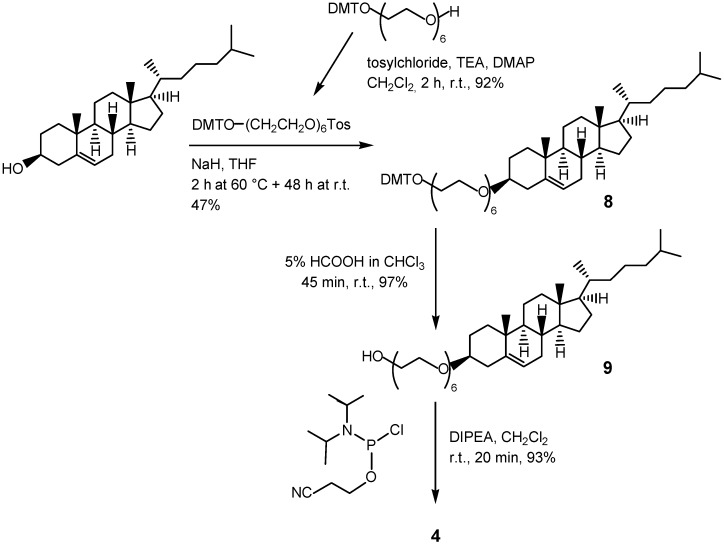
Synthesis of phosphoramidite derivative **4**.

Phosphoramidite derivative **4** has been prepared following the synthetic procedure depicted in Scheme 2. The insertion of the HEG spacer arm onto the steroid backbone has been realized in 47% yield by NaH-promoted condensation of the secondary 3-OH moiety of cholesterol with the tosyl-activated HEG derivative, previously protected at one OH group with the acid-labile DMT group. The successive removal of DMT from **8** with 5% formic acid in CHCl_3_ led in 97% yield to primary alcohol **9**, which was then reacted with chloro-(2-cyanoethoxy)(*N*,*N*-diisopropylamino)phosphine in the presence of DIPEA, giving target compound **4** in 93% yield (42% overall yield for the three reaction steps starting from cholesterol).

All the intermediates and the final compounds have been purified by silica gel chromatography and fully characterized by ^1^H-, ^13^C-, ^31^P- (where present) NMR and ESI-MS analysis. Obtained data for known phosphoramidite **1** are in accordance with the literature values [16].

The synthesis of 5'-conjugated oligonucleotides **A** and **B** has been realized on a dG-CPG support on which the 6-mer d(^5^^'^TGGGAG^3^^'^) has been assembled on a 15 μmol scale by a standard solid phase β-cyanoethyl-phosphoramidite protocol. The on-line coupling of d(^5'^TGGGAG^3'^)-CPG support with both phosphoramidite **1** and **4** was carried out manually, using a standard activator (tetrazole) but prolonged reaction times (2 × 20 min) compared to routine protocols for the solid phase synthesis of oligonucleotides. Notably, as also previously reported [16], phosphoramidite **1** was not very soluble in acetonitrile and its use required the addition of one volume of anhydrous CH_2_Cl_2_ in the coupling step. On the contrary, no solubility problem was observed in acetonitrile with phosphoramidite **4** and a routine coupling protocol could be adopted. After oxidation, the 2-cyanoethyl protecting groups were cleaved from the functionalized solid supports upon reaction with an anhydrous triethylamine/pyridine solution (1:1, v/v) at 50 °C for 2 h [33]. A successive basic treatment with conc. NH_4_OH at 55 °C for 14 h allowed the detachment of the oligonucleotides from the support and the complete removal of the nucleobase protecting groups.

**Figure 4:**
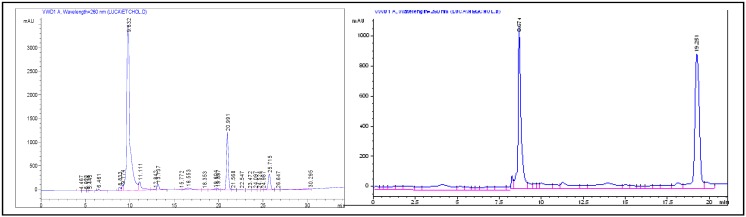
HPLC profiles for the analysis of crude oligomer **A** (left) and **B** (right).

The detached crude oligonucleotides were then analyzed and purified by HPLC on an analytical RP18 column, showing in both cases two main peaks, with retention times of 9.8 and 21.0 min, for oligomer **A**, and 8.7 and 19.2 min for oligomer **B**, respectively (Figure 4) under the same elution conditions (see Experimental Section). As also found by Hotoda and coworkers and by us [26,28], in all the purification attempts on HPLC, the fastest eluting peak, attributed to the single strand 6-mer d(^5^^'^TGGGAG^3^^'^), was accompanied by a peak having a higher retention time. This additional peak was attributed to a G-quadruplex structure formed under the HPLC elution conditions. In fact, by re-injecting the isolated fastest eluting peaks on HPLC, we always observed also the slowest eluting ones; in addition, the two isolated peaks for each crude oligomer, analyzed under the same conditions by MALDI-TOF mass spectrometry, showed very similar *m/z* values for the molecular ions, in accordance with the expected mass, thus supporting their proposed molecular identity [34].

After HPLC purification, the oligonucleotide samples were desalted on a Sephadex G25 column eluted with H_2_O/EtOH (3:1, v/v). The isolated oligomers proved to be more than 98% pure by RP-HPLC analysis and were characterized by MALDI-TOF mass spectrometry.

UV analysis at λ = 260 nm and 90 °C of the final samples dissolved in pure H_2_O showed that very similar isolated yields were obtained for the two compounds: in fact, starting from *ca.* 80 mg of functionalized dG-CPG support for both syntheses, 60 OD of 5'-conjugated oligonucleotide **A** and 58 OD of **B** were obtained.

Analyzing the RP-HPLC behavior of the two conjugated oligomers, compound **B** shows a lower lipophilicity than **A**, as expected. Remarkably, there is also an indication of its higher propensity to form G-quadruplex assemblies, as judged by comparing the ratios of the fastest eluting peak (single strand) *vs.* the slowest eluting peak (G-quadruplex complex), which, even if variable with time and external conditions (*i.e.*, temperature, concentration), was always much higher for **A** than for **B**. This behavior can be attributed to the higher flexibility of the HEG spacer in **B** compared to the mono-ethylene glycol unit in **A**. As a matter of fact, the latter spacer may be not long enough to prevent cholesterol from partially hampering—due to steric hindrance—the G-tetrad formation process at the 5'-end of the G-quadruplex structure. An in-depth biophysical characterization of the G-quadruplex structures generated by **A** and **B** in comparison with the unmodified oligomer, as well as experiments to evaluate their *in vitro* antiviral activity are currently in progress in collaboration with specialized laboratories.

## 3. Experimental

### 3.1. General Methods

All the reagents were of the highest commercially available quality and were used as received. TLC analyses were carried out on silica gel plates from Merck (60, F254). Reaction products on TLC plates were visualized by UV light and then by treatment with an oxidant aq. solution (acetic acid/H_2_O/H_2_SO_4_, 10:4:5, v/v/v). For column chromatography, silica gel from Merck (Kieselgel 40, 0.063–0.200 mm) was used. NMR spectra were recorded on Bruker WM-400, Varian XR 200 and Varian Inova 500 spectrometers, as specified. All the chemical shifts are expressed in ppm with respect to the residual solvent signal. Peak assignments have been carried out on the basis of standard ^1^H-^1^H COSY and HSQC experiments. An Agilent HPLC system (1200 series), equipped with a UV detector, was used for the oligonucleotide analysis and purification. For the ESI MS analyses, a Waters Micromass ZQ instrument—equipped with an Electrospray source—was used in the positive and/or negative mode. MALDI TOF mass spectrometric analyses were performed on a PerSeptive Biosystems Voyager-De Pro MALDI mass spectrometer in the Linear mode, using a mixture of picolinic and 3-hydroxypicolinic acid as the matrix. UV measurements were carried out on a Jasco V-530 UV spectrophotometer equipped with a Jasco ETC-505T temperature controller unit. Abbreviations list: AcOEt = ethyl acetate; *t*-Bu = *tert*-butyl; Chol = cholesterol; CPG = Controlled Pore Glass support; DIPEA = *N*,*N*-diisopropylethylamine; DMAP = *N*,*N*-dimethylaminopyridine; DMT = 4,4'-dimethoxy-triphenylmethyl; Et_2_O = diethyl ether; HEG = hexaethylene glycol; THF = tetrahydrofuran; TEA = triethylamine; TEG = tetraethylene glycol; TEAB = triethylammonium bicarbonate; Tos = *p*-toluene-sulfonate. 

### 3.2. Synthesis of Derivative **5**

Cholesterol (963 mg, 2.49 mmol), dissolved in anhydrous THF (4.0 mL), was treated with *tert*-butyl bromoacetate (0.920 mL, 6.23 mmol) and NaH (200 mg, 60% dispersion in mineral oil, *ca.* 5.0 mmol) at 0 °C. After 24 h at r.t., the reaction was quenched by addition of CH_3_OH (1.0 mL) at 0 °C and then stirred for 10 min. The reaction mixture was next successively concentrated under reduced pressure, diluted with CH_2_Cl_2_ and extracted twice with H_2_O/CH_2_Cl_2_; the organic phases were combined, dried over anhydrous Na_2_SO_4_, filtered and concentrated under reduced pressure. The residue was then purified on a silica gel column, eluted with *n*-hexane/AcOEt 9:1 (v/v), furnishing 392 mg of pure target compound **5** (0.78 mmol, 31% yield). ^1^H-NMR (200 MHz, CDCl_3_): relevant signals at δ 5.36 (d, *J* = 5.0 Hz, 1H, H-6), 4.20 (s, 2H, CH_2_C=O), 3.23 (m, 1H, H-3), 2.31 (m, 2H, CH_2_-4), 1.48 (s, 9H, CH_3_ *t*-Bu), 1.02 (s, 3H, CH_3_-19), 0.91 (d, *J* = 6.5 Hz, 3H, CH_3_-21), 0.86 (overlapped d’s, *J* = 4.5 Hz, 6H, CH_3_-26 and CH_3_-27), 0.67 (s, 3H, CH_3_-18). ^13^C-NMR (50 MHz, CDCl_3_): relevant signals at δ 170.1 (C=O), 140.6 (C-5), 121.8 (C-6), 81.3 (quaternary C of *t*-Bu), 79.8 (C-3), 66.1 (CH_2_C=O), 28.1 (CH_3_*t*-Bu), 22.5 and 22.8 (C-26 and C-27), 19.3 (C-19), 18.7 (C-21), 11.8 (C-18). ESI-MS (positive ions): *m/z* for C_33_H_56_O_3_calcd. 500.4229; found: 522.25 (M+Na^+^), 538.45 (M+K^+^).

### 3.3. Synthesis of Derivative **6**

Derivative **5 **(300 mg, 0.599 mmol), dissolved in CH_2_Cl_2_ (0.75 mL) was treated with HCOOH (3.0 mL) and the resulting mixture was stirred for 12 h at r.t. The reaction solution was taken to dryness under reduced pressure and then coevaporated with CHCl_3_ (3 × 5 mL). Compound **6** was thus obtained in a pure form in an almost quantitative yield (266 mg, 0.598 mmol). ^1^H-NMR (200 MHz, CDCl_3_): relevant signals at δ 5.34 (d, *J* = 4.8 Hz, 1H, H-6), 4.17 (s, 2H, CH_2_C=O), 3.28 (m, 1H, H-3), 2.30 (m, 2H, CH_2_-4), 1.02 (s, 3H, CH_3_-19), 0.91 (d, *J *= 6.5 Hz, 3H, CH_3_-21), 0.86 (overlapped d’s, *J* = 4.5 Hz, 6H, CH_3_-26 and CH_3_-27), 0.68 (s, 3H, CH_3_-18). ^13^C-NMR (50 MHz, CDCl_3_): relevant signals at δ 175.3 (C=O), 140.1 (C-5), 122.2 (C-6), 80.3 (C-3), 65.1 (CH_2_C=O), 22.8 and 22.5 (C-26 and C-27), 19.3 (C-19), 18.7 (C-21), 11.8 (C-18). ESI-MS (positive ions): *m/z* for C_29_H_48_O_3_ calcd. 444.3603; found: 466.72 (M+Na^+^), 482.88 (M+K^+^).

### 3.4. Synthesis of Derivative **7**

Derivative **6** (266 mg, 0.598 mmol), dissolved in anhydrous diethyl ether (4.0 mL) was treated with LiAlH_4_ (45 mg, 1.20 mmol) and the resulting mixture was refluxed for 12 h. Then the solution was diluted with CH_2_Cl_2_ and HCOOH was added dropwise till no evolution of gas was observed. The reaction mixture was successively extracted twice with H_2_O/CH_2_Cl_2_, the organic phases were collected, dried over anhydrous Na_2_SO_4_, filtered and concentrated under vacuum. The residue was then purified on a silica gel column, eluted with *n*-hexane/AcOEt 7:3 (v/v), giving 192 mg of pure derivative **7 **(0.445 mmol, 74% yield). ^1^H-NMR (500 MHz, CDCl_3_): relevant signals at δ 5.34 (d, *J* = 5.0 Hz, 1H, H-6), 3.72 (dd, *J* = 4.0 and 3.3 Hz, 2H, HO-CH_2_CH_2_-O*Chol*), 3.60 (dd, *J* = 3.3 and 3.8 Hz, 2H, HO-CH_2_CH_2_-O*Chol*), 3.23 (m, 1H, H-3), 2.40–2.19 (m, 2H, CH_2_-4), 0.91 (d, *J* = 6.5 Hz, 3H, CH_3_-21), 0.86 (overlapped d’s, *J* = 4.5 Hz, 6H, CH_3_-26 and CH_3_-27), 0.68 (s, 3H, CH_3_-18). ^13^C-NMR (50 MHz, CDCl_3_): relevant signals at δ 140.6 (C-5), 121.6 (C-6), 79.4 (C-3), 69.0 (HO-CH_2_CH_2_-O*Chol*), 61.9 (HO-CH_2_CH_2_-O*Chol*), 22.7 and 22.5 (C-26 and C-27), 19.3 (C-19), 18.7 (C-21), 11.8 (C-18). ESI-MS (positive ions): *m/z* for C_29_H_50_O_2_ calcd. 430.3811; found: 452.65 (M+Na^+^), 468.69 (M+K^+^).

### 3.5. Synthesis of Derivative **1**

Derivative **7** (187 mg, 0.434 mmol), dissolved in anhydrous CH_2_Cl_2_ (2.0 mL) taken over 4Å activated molecular sieves, was treated with DIPEA (230 μL, 1.32 mmol) and chloro-(2-cyanoethoxy)-(*N*,*N*-diisopropylamino)phosphine (115 μL, 0.521 mmol). The resulting solution was stirred at r.t. for 20 min, then concentrated under reduced pressure. The residue was then purified on a silica gel column, eluted with *n*-hexane/AcOEt 7:3 (v/v), furnishing 265 mg of pure phosphoramidite **1** (0.421 mmol, 97% yield), as a mixture of diastereoisomers. ^1^H-, ^13^C- and ^31^P-NMR values for the obtained compound were in agreement with lit. data [16]. ESI-MS (positive ions): *m/z* for C_38_H_66_N_2_O_3_P calcd. 629.4811; found: 630.85 (M+H^+^).

### 3.6. Synthesis of DMT-O-HEG-OTos

DMT-O-HEG-OH [33] (430 mg, 0.737 mmol) was dissolved in anhydrous CH_2_Cl_2_ (4.0 mL) and tosyl chloride (161 mg, 0.844 mmol), TEA (300 μL, 2.16 mmol) and DMAP (4.0 mg, 0.033 mmol) were added to the solution. The reaction mixture, kept under stirring for 2 h at r.t., was extracted twice with H_2_O/CH_2_Cl_2_, the organic phases were collected, dried over anhydrous Na_2_SO_4_, filtered and concentrated under vacuum. The residue was then purified on a silica gel column, suspended in CH_2_Cl_2_ containing a few drops of TEA. Eluting the column with CH_2_Cl_2_/CH_3_OH 97:3 (v/v), 500 mg of the target compound were obtained (0.678 mmol, 92% yield). ^1^H-NMR (200 MHz, CDCl_3_): δ 7.81–6.80 (overlapped signals, 17H, aromatic protons of *DMT *and *Tos*), 4.14 (dd, *J *= 4.6 and 4.8 Hz, -CH_2_O*Tos*), 3.79 (s, 6H, OCH_3_ of *DMT *group), 3.70–3.60 (overlapped signals, 20H, -OCH_2_CH_2_O-), 3.23 (dd, *J* = 5.2 and 5.0 Hz, -CH_2_O*DMT*), 2.43 (s, 3H, -CH_3_ *Tos* group). ^13^C-NMR (100 MHz, CDCl_3_): δ 158.3, 145.0, 144.3, 136.2, 129.9, 129.7, 128.0, 127.9, 127.7, 126.5, 112.9 (aromatic C of *DMT *and *Tos*), 85.8 (quaternary C of *DMT *group), 70.6, 69.1, 68.6, 63.0 (C of *HEG *group), 55.1 (OCH_3_of *DMT *group), 21.5 (-CH_3_ of *Tos* group).

### 3.7. Synthesis of Derivative **8**

Cholesterol (239 mg, 0.618 mmol), dissolved in anhydrous THF (2.0 mL), was reacted with NaH (62 mg, 60% dispersion in mineral oil, *ca.* 1.55 mmol) and DMT-O-HEG-OTos (472 mg, 0.639 mmol). The reaction mixture was stirred at 60 °C for 2 h and then taken at r.t. After 48 h the reaction was quenched by addition of few drops of CH_3_OH till disappearance of gas bubbles. Successively the reaction mixture was concentrated under reduced pressure, diluted with CH_2_Cl_2_ and extracted twice with H_2_O/CH_2_Cl_2_. The organic phases were collected, dried over anhydrous Na_2_SO_4_, filtered and concentrated under vacuum. The residue was then purified on a silica gel column, eluted with *n*-hexane/AcOEt 3:2 (v/v), furnishing 276 mg of pure derivative **8** (0.290 mmol, 47% yield). ^1^H-NMR (500 MHz, CDCl_3_): relevant signals at δ 7.46-6.80 (overlapped signals, 13H, aromatic protons of *DMT* group), 5.33 (d, *J *= 5.0 Hz, 1H, H-6), 3.78 (s, 6H, OCH_3_ of *DMT* group), 3.66–3.61 (overlapped signals, 22H, -OCH_2_CH_2_O-), 3.22 (t, *J* = 5.0 and 5.0 Hz, -CH_2_O*DMT*), 3.16 (m, 1H, H-3), 2.37–2.18 (m, 2H, CH_2_-4), 0.99 (s, 3H, CH_3_-19), 0.91 (d, *J* = 6.5 Hz, 3H, CH_3_-21), 0.86 (coincident d’s, *J* = 6.5 Hz, 6H, CH_3_-26 and CH_3_-27), 0.67 (s, 3H, CH_3_-18). ^13^C-NMR (50 MHz, CDCl_3_): relevant signals at δ 158.2, 144.9, 136.2, 129.8, 128.0, 127.9, 127.7, 126.9, 126.4, 112.8 (aromatic carbons of *DMT* group), 140.7 (C-5), 121.3 (C-6), 85.7 (quaternary C of *DMT* group), 79.2 (C-3), 70.4, 67.1, 62.9, 60.1 (C of *HEG *group), 54.9 (OCH_3_ of *DMT* group), 22.7 and 22.4 (C-26 and C-27), 19.2 (C-19), 18.6 (C-21), 11.7 (C-18). ESI-MS (positive ions): *m/z* for C_60_H_88_O_9_ calcd. 952.6428; found: 975.18 (M+Na^+^), 991.16 (M+K^+^).

### 3.8. Synthesis of Derivative **9**

Derivative **8** (245 mg, 0.257 mmol) was dissolved in 5% HCOOH solution in CHCl_3_ (3.0 mL) and the resulting mixture was stirred for 45 min at r.t. Then the reaction mixture was concentrated and purified on a silica gel column, eluted with CHCl_3_/CH_3_OH 98:2 (v/v), which furnished 162 mg of pure derivative **9** (0.249 mmol, 97% yield). ^1^H-NMR (500 MHz, CDCl_3_): relevant signals at δ 5.34 (d, *J *= 5.0 Hz, 1H, H-6), 3.82–3.61 (overlapped signals, 24H, -OCH_2_CH_2_O-), 3.18 (m, 1H, H-3), 2.37–2.18 (m, 2H, CH_2_-4), 1.01 (s, 3H, CH_3_-19), 0.91 (d, *J* = 6.5 Hz, 3H, CH_3_-21), 0.86 (coincident d’s, *J* = 4.5 Hz, 6H, CH_3_-26 and CH_3_-27), 0.67 (s, 3H, CH_3_-18). ^13^C-NMR (100 MHz, CDCl_3_): relevant signals at δ 140.9 (C-5), 121.4 (C-6), 79.4 (C-3), 72.4, 70.5 and 67.2 (C of *HEG *group), 22.7 and 22.4 (C-26 and C-27), 19.3 (C-19), 18.6 (C-21), 11.7 (C-18). ESI-MS (positive ions): *m/z* for C_39_H_70_O_7_ calcd. 650.5122; found: 673.65 (M+Na^+^), 689.56 (M+K^+^).

### 3.9. Synthesis of Derivative **4**

To derivative **9** (150 mg, 0.231 mmol), dissolved in anhydrous CH_2_Cl_2_ (2.0 mL) taken over 4 Å molecular sieves, DIPEA (140 μL, 0.804 mmol) and chloro-(2-cyanoethoxy)(*N*,*N*-diisopropyl-amino)phosphine (68 μL, 0.307 mmol) were sequentially added. The resulting solution was kept under stirring for 20 min, then concentrated under reduced pressure and the corresponding residue purified on a silica gel column eluted with AcOEt, which gave 183 mg of pure phosphoramidite **4** (0.215 mmol, 93% yield), as a mixture of diastereoisomers. ^1^H-NMR (400 MHz, CDCl_3_): relevant signals at δ 5.26 (d, *J* = 5.0 Hz, 1H, H-6), 3.76–3.30 (overlapped signals, 28H, -OCH_2_CH_2_O-, -N[CH(CH_3_)_2_] and -OCH_2_CH_2_CN), 3.10 (m, 1H, H-3), 2.73 (broad signal, 2H, -CH_2_CH_2_CN), 2.31–2.28 (m, 2H, CH_2_-4), 0.92 (overlapped signals, 6H, -N[CH(CH_3_)_2_]), 0.83 (overlapped d’s, *J* = 4.5 Hz, 6H, CH_3_-26 and CH_3_-27), 0.60 (s, 3H, CH_3_-18). ^13^C-NMR (100 MHz, CDCl_3_): relevant signals at δ 140.8 (C-5), 121.3 (C-6), 117.5 (CN), 79.3 (C-3), 70.7, 69.8, 67.1 (C of *HEG *group), 56.5 (-OCH_2_CH_2_CN), 42.8 (-N[CH(CH_3_)_2_]), 23.6 (-N[CH(CH_3_)_2_]), 22.6 and 22.4 (C-26 and C-27), 20.8 (-OCH_2_CH_2_CN), 19.2 (C-19), 18.5 (C-21), 11.7 (C-18). ^31^P-NMR (CDCl_3_, 161.98 MHz): δ 149.1 and 149.0. ESI-MS (positive ions): *m/z* for C_48_H_86_N_2_O_8_P calcd. 849.6122; found: 850.93 (M+H^+^); 872.60 (M+Na^+^).

### 3.10. Solid Phase Synthesis of Oligonucleotides **A** and **B**

Two batches of CPG-^3'^GAGGGT-DMT^5'^ resin (*ca.* 80 mg each) with 0.030 mmol/g initial functionalization were detritylated with a 10% TCA solution in CH_2_Cl_2_ and then exhaustively washed with anhydrous acetonitrile. On each of them, two coupling cycles, of 20 min each, were carried out with either phosphoramidite derivative **1** or phosphoramidite derivative **4**, respectively (0.02 mmol per coupling) dissolved in 600 μL of a 0.45 M tetrazole solution in CH_3_CN/CH_2_Cl_2_ 1:1 (v/v) for **1**, and 0.45 M tetrazole solution in CH_3_CN, for phosphoramidite **4**. The coupling was followed by a 20 min standard oxidation step using a 0.02 M solution of I_2_ in Py/H_2_O/THF. After each step, the solid supports were exhaustively washed with anhydrous CH_3_CN and then treated with a triethylamine/pyridine solution (1:1, v/v) at 50 °C for 2 h. Subsequent treatment with conc. aq. NH_4_OH for 14 h at 55 °C allowed to release the fully deprotected oligonucleotides in solution.

### 3.11. Purification and Characterization of the 5'-Conjugated Oligonucleotides

The crude oligonucleotides **A** and **B** were purified by HPLC on an analytical reverse phase column (PHENOMENEX^®^ 100-5 C18), using as eluent a linear gradient from 0% to 100% of CH_3_CN in 0.1 M TEAB in 20 min (flow rate 0.8 mL/min, detection at λ = 260 nm). In both cases, the HPLC profiles showed two main peaks.

For oligonucleotide **A**, the peaks with 9.8 min and 21.0 min retention time were collected and concentrated [34].The isolated compounds were then desalted on a Sephadex G25 column eluted with H_2_O/EtOH (3:1, v/v). The fractions showing absorbance at λ = 260 nm were collected and taken to dryness, furnishing 41 OD and 19 OD for the samples corresponding, respectively, to the compounds with the lowest and highest HPLC retention time. The two samples isolated from crude oligonucleotide **A** were then analyzed by MALDI-TOF MS in the negative ions mode. Very similar *m/z* values were found for the molecular ions, thus furnishing a clear experimental evidence in support of their structural identity.

MALDI: calcd. for C_89_H_124_N_27_O_40_P_7_: 2427.67; *m/z*, found for the sample with retention time 9.8 min: 2425.81 (M-H^+^); *m/z*, found for the sample with retention time 21.0 min: 2426.18 (M−H^+^).

HPLC analysis showed two peaks, with respectively 8.7 and 19.2 min retention times, also for crude oligonucleotide **B**, which were collected and concentrated, then desalted on a Sephadex G25 column eluted with H_2_O/EtOH (3:1, v/v). The fractions showing absorbance at λ = 260 nm were concentrated under reduced pressure, furnishing, respectively, 35 OD for the fastest and 23 OD for the slowest eluting peak. Also in this case, MALDI-TOF MS analysis of the two isolated species showed data fully supporting their identity.

MALDI: calcd. for C_99_H_144_N_27_O_45_P_7_: 2647.80; *m/z*, found, for the sample with retention time 8.7 min: 2647.09 (M−H^+^); *m/z*, found for the sample with retention time 19.2 min: 2647.47 (M−H^+^).

## 4. Conclusions

With the aim of expanding the repertoire of available lipid-based phosphoramidite building blocks, useful to convert biologically active oligonucleotide sequences into therapeutically viable drugs, we have described the synthesis and characterization of a novel phosphoramidite derivative of cholesterol, and compared its use to a previously described analog, differing in the length of the spacer arm (hexa- *vs.* mono-ethylene glycol). Straightforward procedures, exploiting only stable, inexpensive and common chemicals, allowed us to convert—in a few, highly reproducible steps—the starting cholesterol into the target derivatives. To test their applicability on G-quadruplex-based aptamers, these building blocks have been inserted at the 5'-end of the d(^5'^TGGGAG^3'^) sequence using a standard phosphoramidite, on-line conjugation protocol, in order to obtain new potential anti-HIV agents. The synthesized cholesteryl conjugated oligonucleotides have been purified by HPLC and characterized through MALDI-MS analysis.
